# Artificial intelligence in imaging flow cytometry

**DOI:** 10.3389/fbinf.2023.1229052

**Published:** 2023-10-09

**Authors:** Paolo Pozzi, Alessia Candeo, Petra Paiè, Francesca Bragheri, Andrea Bassi

**Affiliations:** ^1^ Department of Physics, Politecnico di Milano, Milano, Italy; ^2^ Institute for Photonics and Nanotechnologies, Consiglio Nazionale delle Ricerche, Milano, Italy

**Keywords:** cytometry, imaging flow cytometry, optofluidcs, high content screening, lab on a chip (LoC)

## Introduction

Imaging flow cytometry (IFC) is a powerful screening technique that combines the advantages of flow cytometry and optical microscopy (Basiji et al., 2007; Rees et al., 2022). By capturing microscopy images of the specimens as they move along a liquid stream, IFC provides high-throughput collection of morphological and spatial information from thousands or even millions of samples. This makes it a key enabling technology for screening at the single-cell level, which is fundamental for identifying and characterizing pathogenic drivers and biomarkers in a cellular population and for understanding heterogeneity in a biological system.

Imaging flow cytometry can be used at different scales, to study bioparticles such as extracellular vesicles (Lannigan and Erdbruegger, 2017; Görgens et al., 2019), bacteria (Power et al., 2021) and cells. It is widely used to study complex tissues, by dissociating the specimen in single cells (Covarrubias et al., 2019). New imaging systems, combined with custom microfluidics are opening to the study of entire organisms, including *C. Elegans* (Hernando-Rodríguez et al., 2018), *Drosophila* (Memeo et al., 2021) and zebrafish (Liu et al., 2017) or organoids (Paiè et al., 2016) in three dimensions.

Imaging flow cytometry is today a tool for biological, drug discovery and clinical research. It has the potential to transform into a clinical diagnostic method (Doan et al., 2018), but advancements are needed both in automation and in artificial intelligence to handle and analyze the large amount of data retrieved by such high-throughput methods. In this paper we will introduce the typical pipelines for IFC acquisition and processing, and we will focus on the challenges that artificial intelligence should address to facilitate the transformation of IFC from a scientific to a medical diagnostic tool.

### Data acquisition schemes

**FIGURE 1:**
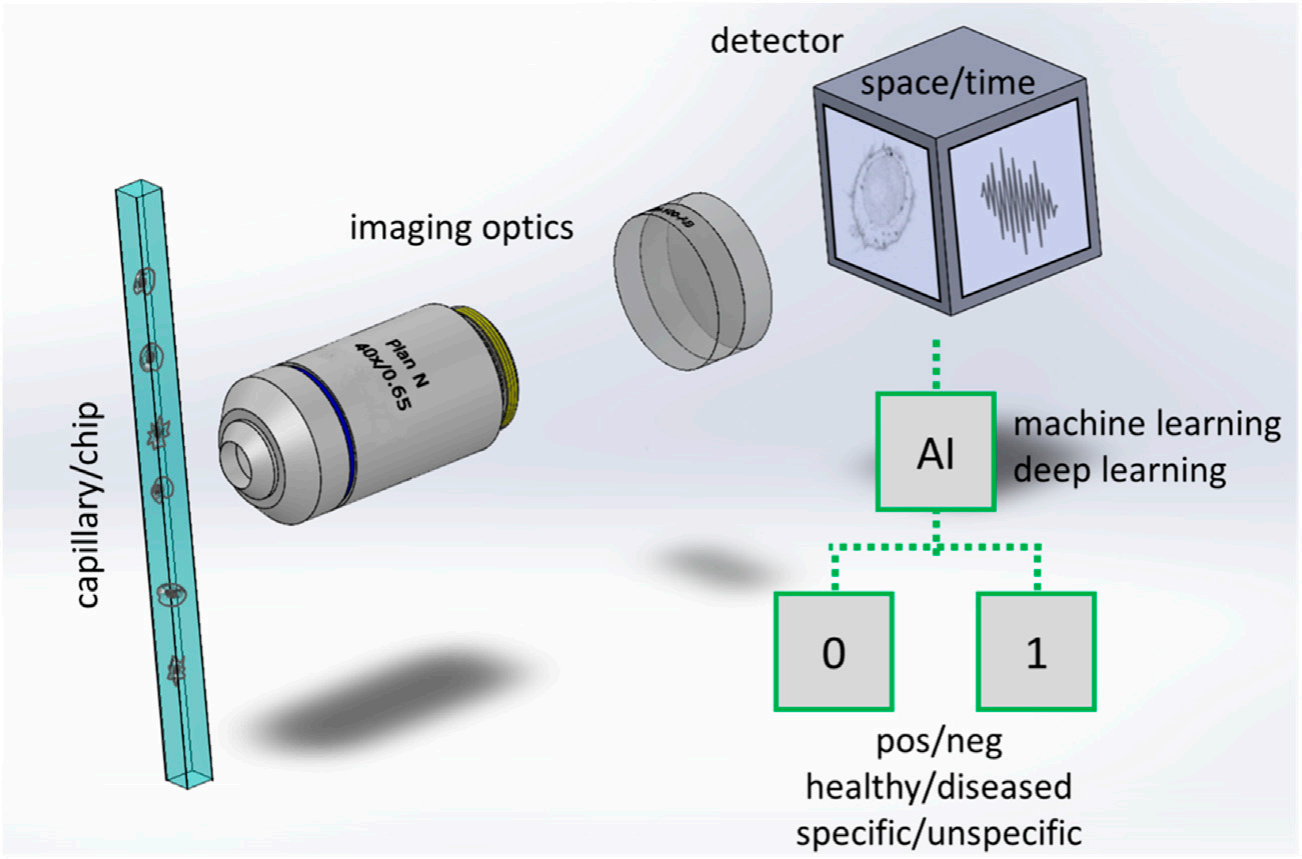
Scheme of an imaging flow cytometer with workflow for binary classification. Cells or particles are rapidly flowing in a capillary or in a more advanced lab on chip that has at least a transparent window for collecting the light emitted or transmitted by the samples. In a typical implementation a microscope consisting of high magnification optics (an objective lens and a tube lens are shown) forms the image of the cell/particles at a widefield detector. In other implementations (e.g., Ota et al., 2018) the images are not directly acquired in space, but a time signal is collected by a single pixel detector while the samples are passing through a specialized illumination. Artificial intelligence is used to process the images or the time signal, typically to classify the cells/particles in two (binary classification) or more classes.

In an IFC system, the specimen flows in a capillary and images of the specimen are rapidly captured by a detector (Figure 1). Illumination of the sample is provided by light emitting diodes (LED) or lasers (not shown in the figure) in different configurations, which include transillumination (Basiji et al., 2007), excitation from an angle (Goktas et al., 2019), structured (Mashayekh et al., 2022) or light-sheet illumination (Gualda et al., 2017). The samples flow in a simple straight tube, in more sophisticated capillaries, or in lab-on-chip devices (Paiè et al., 2018). The detection is typically performed with widefield cameras (CCD or fast CMOS cameras) but single pixels detection methods such as Ghost cytometry (Ota et al., 2018) or methods encoding spatial information into spectral or temporal codifications (Wu et al., 2017; Mikami et al., 2018) are showing potential guaranteeing high speed rates, good image resolution as well as continuative acquisition modality, particularly in combination with computational imaging and machine learning methods (Ota et al., 2020).

Different acquisition modes are adopted in IFC, including brightfield, darkfield, phase contrast, and fluorescence (Basiji et al., 2007). Morphological and functional information can be extracted exploiting these contrast mechanisms; even mechanical phenotyping can be assessed using in-flow deformability measurements allowing the quantification of physiological and pathological functional modifications (Otto et al., 2015; Soteriou et al., 2023).

In fluorescence-IFC, the development of light sheet fluorescence microscopy (LSFM) has recently opened new possibilities. LSFM uses a thin sheet of light to excite only the fluorophores within a restricted focal volume, providing optical sectioning capability to the imaging system, and uses widefield cameras to record hundreds or thousands of images of the samples per second. This high acquisition speed and high collection efficiency have facilitated the development of IFC at extremely high throughput (Holzner et al., 2021; Ugawa and Ota, 2022), and the implementation of 3D screening methods (Sala et al., 2020; Vargas-Ordaz et al., 2021), which can even be used to study entire organisms (Memeo et al., 2021; Bernardello et al., 2022), without dissociating the tissue in single cells. The adoption and further development of three-dimensional imaging will likely contribute to improving the study of proteins localization, co-localization, and to protein-protein interactions within the entire sample volume.

Among label free IFC stands out Quantitative Phase Imaging (QPI), as it enhances the acquired sample information, enabling the acquisition of the optical phase shift induced by the sample, which is given by both the sample thickness and the optical refractive index (Bianco et al., 2017). Three dimensional QPI enables decoupling these parameters and obtaining three-dimensional refractive index reconstruction, by illuminating the sample along different directions. Recently, this method has been successfully coupled with microfluidic sample delivery exploiting the self-rotation of cells in fluids (Villone et al., 2018; Pirone et al., 2022). However, these developments in throughput and multidimensional imaging come with an increased amount of acquired data, opening new challenges in real time and quantitative image processing.

### Real time processing

Computer vision algorithms are routinely used in processing conventional two-dimensional IFC data. These allow the identification of the flowing samples, the extraction of signal from background, and cells segmentation (Pedreia et al., 2013). Segmentation of isolated single cells in a fluidic system is efficiently achieved with standard image processing pipelines, based, for example, on local thresholding and binary morphological operations. While these approaches work efficiently in two-dimensional IFC, they become less accurate when considering 3D data, such as cells aggregates, tissue chunks or entire organisms.

In this context, convolutional neural networks can produce higher quality results, especially for complex samples, and can be less dependent on the segmentation parameters chosen by manual operators. Machine learning models (Henning et al., 2017) are being developed to process the images rapidly and are becoming the tool of choice for segmentation in advanced IFC systems. Deep learning opens the way to semantic segmentation of complex cells or structures, which consists in associating a label or category to every pixel of the sample image.

Machine and deep learning are increasingly used in IFC, a number of architectures have been exploited (for a review see Luo et al., 2021). The need for machine and deep learning tools is further emphasized by the fact that segmentation is only a preprocessing step in IFC. Real time sample classification and labeling is required in the majority of the screening applications, for different reasons:

(i) In cell sorting devices, the IFC system is used to distinguish a particular cell type from others contained in a sample on the basis of a specific label. The cells flowing in a capillary or in a fluidic chip are physically separated by an actuator that divides them into multiple batches. Image processing should permit the labeling in milli to micro-seconds time scales.
(ii) Ideally in a diagnostic system the samples should be classified into two classes (healthy/unhealthy as in Figure 1) or in multiple classes, indicating e.g., the state of a certain disease, the level of inflammation or to identify the presence of heterogeneities in the tissue.
(iii) In future portable systems and point-of-care devices, the storage of large amounts of raw data could be critical. Segmentation, classification, and labeling should be performed in real time, in order to retrieve and store the screening results only. However, this requires the development of very accurate and reliable processing methods.

Deployment of real-time machine learning and deep learning algorithms is possible in different hardware configurations. While Central Processing Units (CPUs) and Graphics Processing Units (GPUs) are well suited for offline operation, prototypes based on Field Programmable Gate Arrays (FPGA) have shown promising results for the future development of Application Specific Integrated Circuits (ASIC), with the potential to allow real time processing in miniaturized devices with minimal power requirements (Isozaki et al., 2019a). A possible emerging alternative is the use of System on a Chip (SoC) that integrates GPU and CPU in the same electronic chip.

The hardware for analyzing IFC data will undoubtedly progress in the next years, and we do not expect it to be the limiting factor in the deployment of real-time analysis pipelines. Nevertheless, IFC data processing opens new challenges related to the robustness of machine learning and deep learning approaches. Robustness refers to the ability of an AI algorithm to maintain its performance and accuracy even in the presence of unforeseen or unexpected input or environmental changes. In the context of IFC, a robust AI system can handle image artifacts, optical aberrations, staining variations in cell, low signal-to-noise ratio, and continues to perform the required tasks reliably and efficiently. Achieving robustness is important in building all AI systems, but it is critical in medical diagnosis. We delve here into two aspects that are, in our opinion, particularly relevant to the robustness of machine learning and deep learning methods in image-based flow cytometry: training and standardization.

### Training

While the identification (Yang et al., 2018) or compensation (Guo et al., 2020) of image artefacts and aberrations due to imperfections of the acquisition system can be corrected with general purpose machine learning methods, machine learning-based analysis for imaging flow cytometry starts from building a training dataset and training a model on that dataset. These steps rely critically on accurate data annotation.

Manual annotation is the traditional method of labeling data, but it can be time-consuming in the context of IFC, considering that a massive amount of data can be acquired. It becomes a labor-intensive process that limits the scalability of machine-learning-based approaches to IFC analysis. In addition, manual operators may not be capable of accurately discriminating healthy from diseased cells or classifying different cell types and states. Automating the annotation process is essential for improving machine learning-based approaches.

Fluorescence imaging can facilitate data annotation, as the fluorescence signal can often be associated with a particular cell state, providing a strong supervision that does not require manual labeling of individual images. This linking of fluorescence to the state of cells and to the presence of different biomarkers is an available option, employed in biological screening and clinical research (Refaat et al., 2022). However, fluorescence has its drawbacks when considering its use in diagnostics, as it complicates the sample preparation workflow, and it increases the preparation time (incubations are needed for cellular staining with fluorescent markers). This makes it less appealing from a clinical perspective.

Label-free approaches are very attractive, instead. One can identify protocols where the cells are extracted from a liquid biopsy, or disaggregated from solid biopsies, and are directly processed without staining. Brightfield, phase and scattering measurements offer faster and simpler sample preparation workflows. Yet, one of the challenges in cell classification from label free imaging is how to accurately identify and classify a cell when specific labeling is unavailable.

The observation of cell morphology has a significant potential for distinguishing between different cell types and states and for identifying various diseases, being the pilasters of classic cytological differential diagnosis. This potential is emerging in one field that is particularly relevant for diagnosis, i.e., automatic screening of blood samples. Bright-field and dark-field IFC have been implemented to identify phases in the cell cycle (Eulenberg et al., 2017) and classify white blood cell types (Nassar et al., 2019; Lippeveld et al., 2020). Similarly, IFC was used for acute lymphoblastic leukemia diagnostics, using a residual convolutional neural network (CNN) architecture (Doan et al., 2020a, Cytometry Part A). Morphology based identification has also been used to differentiate aggregated platelets from single platelets and white blood cells with a high specificity (Jiang et al., 2017). Doan et al (Doan et al., 2020b, PNAS) used IFC and deep learning to distinguish clinically relevant red blood cells morphologies associated with cell storage lesions. In the field, deep learning is expected to be applicable to many other medical image classification tasks (Rubin et al., 2019).

To avoid manual annotation, researchers have explored innovative approaches, such as weakly supervised machine learning models (Zhou, 2018) that can learn to associate IFC data with macroscopic biological and clinical variables. A notable example was developed for the diagnosis of Sézary syndrome. The training was based on the information about the disease state, at the level of the specimen, which was extracted from the entire collection of cells images (Otesteanu et al., 2021). Although this approach requires clear morphological manifestations in the malignant cells, its generalization is possible. Generalization is facilitated when there is a morphological similarity between the specimens used for training the model and the specimens derived when investigating a new pathology. Nevertheless, the use of transfer learning methods could play a substantial role in accelerating the adoption of self and weakly supervised deep learning that will circumvent the need for manual labelling.

### Standardization

A prerequisite to the development of robust AI pipelines, is the establishment of standard calibration, acquisition, and processing protocols. In the field of IFC, these protocols are lacking partially because the technique is not widely adopted yet and because custom scientific instruments are constantly developed, employing different and novel imaging modalities and lab-on-chip devices.

Standardization helps to ensure consistency and reproducibility in acquisition and processing of data collected across different samples, experiments, and laboratories. At the same time, establishing guidelines with consistent steps for sample preparation, image pre-processing, cell segmentation, feature extraction, and data annotation, can reduce potential sources of variability and errors. Finally, standard pipelines provide a reference point for validating and benchmarking new AI algorithms or for improving the existing ones. In the fields of flow cytometry and microscopy, two initiatives (Quarep-LiMi and MIFlowCyt) are supporting the establishment of standardized guidelines and protocols.

QUAREP-LiMi, Quality Assessment and Reproducibility for Instruments and Images in Light Microscopy (Nelson et al., 2021), provides a set of guidelines for assessing the quality and reproducibility in light microscopy. It covers aspects such as instrument setup, data acquisition and analysis, and reporting standards. The aim is to improve the reliability and comparability of microscopy data across different laboratories.

MIFlowCyt, The Minimum Information about a Flow Cytometry Experiment (Lee et al., 2008), consists of a set of guidelines that ensure that critical information is included when reporting a flow cytometry experiment, such as sample preparation, instrumentation, and data analysis. The adoption of MIFlowCyt has facilitated the discovery and reuse of flow cytometry data across different research groups.

The establishment of similar working groups, which involve scientific, industrial, and medical actors, along with regulatory bodies, is urgent to initiate the definition of standard guidelines for calibration, acquisition, processing and use of AI in imaging flow cytometry and cellular screening. Nonetheless such initiative would integrate well with the mandate of the National Institute of Health (NIH) to promote the sharing of scientific data and with the open science policy of the European Commission.

It is worth noting that the diagnostic decision will not be based on a single-cell basis but rather by a combination of the single-cell results, integrating multiple acquisition modalities and even different experimental techniques. This approach is already emerging in cytometry and single-cell acquisition techniques (Ashhurst et al., 2022; Pedersen et al., 2022). Standardization will be crucial in this context, serving as a prerequisite for correct data integration.

In summary, improving the robustness of AI systems is a critical challenge that requires the establishment of clear standards and guidelines for acquisition, processing and AI training. The lack of standards in IFC can limit the progress in the field, but the initiatives started in microscopy and flow cytometry could be the optimal starting point to promote the development of more robust and reliable data-driven IFC systems.

## Conclusion

The integration of AI with cellular screening can revolutionize the way we analyze and understand cells and tissues, leading to new discoveries, and improvements in healthcare. In this setting, IFC has been proved a powerful tool for high-throughput characterization of biomarkers, with its unique asset of enabling an exhaustive sample heterogeneity investigation. Moreover, IFC can evolve into a reliable diagnostic technology that would be a game changer for several diseases, as in the case of liquid biopsy for tumor diagnosis and monitoring. With respect to tissue biopsy, which represents the current gold standard in oncology, it promises non-invasiveness, rapidity, and automation of the analysis. Indeed, microfluidics and lab-on-chips offer the advantage of reducing the cost and complexity of the technology. With high resolution single-cell imaging methods, the data extracted from the sample is maximized, meaning that only small amounts of potentially costly samples are necessary. Moreover, scale-out (i.e., parallelization) of microfluidic systems allows for both small-scale, point-of-care implementation, as well as large-scale, high throughput analyses.

Nevertheless, progress in hardware and software for addressing these new challenges is under the scientific community magnifying glass. Machine learning is poised to play a critical role in the analysis of IFC data, particularly for the segmentation and classification of complex cellular structures. While there are still challenges to be addressed, such as the need for more efficient annotation methods and standardized imaging protocols, the potential benefits of machine learning and deep learning in IFC are clear and are likely to drive further advances in this field in the future years.
